# Characterization of Buritirana (*Mauritiella armata*) Fruits from the Brazilian Cerrado: Biometric and Physicochemical Attributes, Chemical Composition and Antioxidant and Antibacterial Potential

**DOI:** 10.3390/foods11060786

**Published:** 2022-03-09

**Authors:** Florisvaldo Gama de Souza, Fábio Fernandes de Araújo, Eduardo Adilson Orlando, Fernando Morais Rodrigues, Davy William Hidalgo Chávez, Juliana Azevedo Lima Pallone, Iramaia Angélica Neri-Numa, Alexandra Christine Helena Frankland Sawaya, Glaucia Maria Pastore

**Affiliations:** 1 Department of Food Science, Faculty of Food Engineering, University of Campinas, Campinas 13083-862, SP, Brazil; fabio.fernandesn18@gmail.com (F.F.d.A.); eduardo@fea.unicamp.br (E.A.O.); jpallone@unicamp.br (J.A.L.P.); iramaianuma@gmail.com (I.A.N.-N.); glaupast@unicamp.br (G.M.P.); 2 Department of Food Science and Technology, Federal Institute of Education, Science and Technology of Tocantins, Paraíso of Tocantins 77600-000, TO, Brazil; fernandomorais@ifto.edu.br; 3 Department of Food Science and Technology, Federal Rural University of Rio de Janeiro, Seropédica 23890-000, RJ, Brazil; davyhw76@gmail.com; 4 Faculty of Pharmaceutical Sciences, Institute of Biology, University of Campinas, Campinas 13083-862, SP, Brazil; alexandra.sawaya@fcf.unicamp.br

**Keywords:** antimicrobial activity, bioactive compounds, carbohydrate profile, food fibers, functional potential, nutritional composition, phenolic compounds, proximate composition, volatile organic compounds

## Abstract

The buritirana is a little-explored species of the Arecaceae family. The biometric and physicochemical characteristics, nutritional and chemical composition and antioxidant and antibacterial potential of the buritirana fruit fractions were evaluated here for the first time. The fruits presented an oblong shape. The pulp represented 16.58% of the whole-fruit weight (10.07 g). The moisture, ash and soluble fiber contents were similar for the whole fraction without seed (WS) and pulp. Although the total carbohydrate content was the same for seed and peel (23.24 g·100 g^−1^), the seed showed higher protein and insoluble fiber contents. Except for glucose (1256.63 mg·100 g^−1^), the seed showed the highest concentrations of mono-, di- and oligosaccharides. Mineral content ranged from 0.43 to 800 mg·100 g^−1^ in all fractions. The peel fraction showed the highest content of vitamin C. The physicochemical results indicate the pulp and WS fraction have potential for the production of fruit-derived food products. Protocatechuic and quinic acids and epicatechin/catechin were found in all fractions. The assay antioxidant capacity DPPH, phenolic content and total flavonoids were higher in the pulp; TEAC and ORAC_HF_ values were lower in the seed. Volatile organic compounds were not identified, and the fractions did not show antibacterial activity.

## 1. Introduction

For many years, Brazil has attracted the attention of researchers around the world due to its native plant biodiversity. Many of these plants have bioactive substances that have been associated with the prevention and treatment of diseases [1,2].

The Brazilian flora is rich in native palm species with socioeconomic, nutritional and functional importance, and they can be found in areas of transition between the Cerrado, Amazon and Caatinga biomes. Some of these palm trees, such as the oil palm (*Elaeis oleifera*) and açaí (*Euterpe oleraceae*), have been commercially exploited, while buritirana (*Mauritiella armata* Mart.) is still underutilized and needs further studies to promote its cultivation and commercialization [3,4].

*Mauritiella armata* is a species of the Arecaceae family popularly known as buritirana, buriti bravo, yumuna aguajillo and buriti mirim [5]. The fruits of this species are morphologically similar to those of the buriti (*Mauritia flexuosa*), but smaller in size. Its climacteric fruits are drupes round to oval in shape, arranged in clusters, and are covered with small peels that look like overlapping fish scales of a light-orange color. They have a fibrous mesocarp with soft pulp, which varies from yellow to light green and covers a very thin endocarp containing a very hard seed [4,6]. Its fruits are generally marketed and consumed *in natura*, or their pulp is used to produce sweets, jams, beverages, creams, wines and other food products [7].

In local communities, buritirana is used for the treatment of skin burns and rheumatism [8], probably due to the presence of phenolic compounds, which have a high antioxidant capacity and can be effective in combating various diseases [2,9]. Thus, studies on the buritirana are necessary to locate the phenolic compounds, assess their antioxidant capacity and understand the potential benefits of this species.

Few studies have been carried out on the buritirana fruits. Anunciação et al. [10] found some carotenoids in them, such as all-trans-β-carotene (373.00 μg·100 g^−1^), all-trans-α-carotene (230.00 μg·100 g^−1^), all-trans-lutein (198.00 μg·100 g^−1^) and 9-cis-β-carotene (11.00 μg·100 g^−1^); and Ruiz and Villena [11] reported the significant presence of lipids (21.01%), proteins (2.15%), carbohydrates (21.62%) and calories (284.15 Kcal). Souza et al. [12] evaluated the oil extracted from the fruits by supercritical CO_2_ and found high yield, total carotenoids, saturated and unsaturated fatty acids, α-tocopherol and antioxidant capacity. However, no work has assessed the chemical composition and nutritional, biological or technological potential of the buritirana fruit and its fractions. Therefore, this is the first study carried out to determine the biometric and physicochemical characteristics, proximate composition, mineral content, carbohydrate and phenolic compounds profiles, volatile organic compounds, antioxidant capacity and antibacterial activity of buritirana and its fractions.

## 2. Materials and Methods

### 2.1. Chemicals and Reagents

The following chemicals and reagents were purchased from Sigma-Aldrich (St. Louis, MO, USA): Trolox (6-hydroxy-2578-tetramethylchroman-2-carboxylic acid), 2,2-diphenyl-1-picrylhydrazil (DPPH), 2,2′-azobis (2-methylamidinopropane)-dihydrochloride (AAPH), 2,2′-azinobis-(3-ethylbenzothiazoline-6-sulfonic acid)-diammonium salt (ABTS), fluorescein, Folin–Ciocalteu reagent, methanol and formic acid grade HPLC, quinic acid (PubChem CID: 6508) and all phenolic compound standards (protocatechuic acid (PubChem CID: 72), catechin (PubChem CID: 9064), chlorogenic acid (PubChem CID: 1794427), epicatechin (PubChem CID: 72276), p-coumaric acid (PubChem CID: 637542), sinapic acid (PubChem CID: 637775), ferulic acid (PubChem CID: 445858), rutin (PubChem CID: 5280805), quercetrin (PubChem CID: 5280459) with a purity of ≥96%. The other solvents and reagents used in this study were of analytical grade. All solutions were prepared with ultra-pure water (18 MΩ·cm^−1^) obtained from a Milli-Q water purification system (Millipore, Bedford, UK).

### 2.2. Samples

Buritirana fruits in the physiological maturation stage were harvested from 20 plants in November 2018 at Fazenda Moreira (10°33′11″ S; 48°43′50″ W), located in Porto Nacional, TO, Brazil. The fruits were harvested in the afternoon. An exsicate (access number 203,433) was identified and deposited in the Herbarium UEC of the Institute of Biology of the State University of Campinas (UNICAMP), Brazil. The fruits collected were washed and dried in open air at room temperature and then packed in polyethylene packaging for 3 days to ripen completely. Then, the fractions were obtained by the manual pulping of the fruits using stainless steel knives; they were named as follows: WS (whole without seed), PU (pulp), PE (peel) and SE (seed). The fractions were frozen at −20 °C, freeze-dried for 72 h (LIOTOP, model L101, São Carlos, Brazil) and ground using a knife grinder (Marconi, model MA340, Piracicaba, Brazil). They were stored in dark polyethylene bags at −40 °C until the moment of preparing the extracts and carrying out the analyses.

### 2.3. Biometric Characteristics

**Figure 1:**
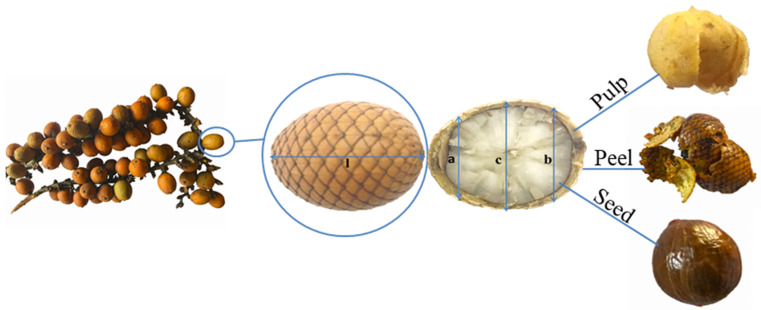
Buritirana fruit (*Mauritiella armata*). (l) Length; (b) basal, (c) central and (a) apical width.

Twenty fruits obtained from different trees were individually weighed on a semi-analytical balance to obtain the average of the total mass of the fruit. With the aid of a digital caliper, the length (l) and the basal (b), central (c) and apical (a) widths of each fruit were measured as illustrated in Figure 1. After manual pulping of each fruit, the pulp, peel and seed fractions were weighed in a semi-analytical balance. The percentage of their fractions was calculated as the ratio between the pulp, peel and seed masses, respectively, and the total mass of the fruit multiplied by 100.

### 2.4. Proximate Composition, Physicochemical Characterization and Vitamin C Content

The freeze-dried fruit fractions were subjected to analyses of protein, dietary fiber, ash, moisture and pH according to standard methods [13]. The carbohydrate content and total titratable acidity (TTA) were determined according to the 040/IV and the 312/IV methods [14], and the total fat content was determined according to [15]. The vitamin C content was assessed according to [16]. The total energy value was estimated using the conversion factors of 4 kcal·g^−1^ for protein or carbohydrate content, 9 kcal·g^−1^ for the lipid content and 2 kcal·g^−1^ for dietary fiber content: Energy (kcal) = [4 × (g protein + g carbohydrate) + 9 × (g lipid) + 2 × (g fiber)] [17]. The total soluble solids (TSS) and the ratio of TSS/TTA [13] were assessed only for WS and PU fractions. Thus, for PE and SE fractions, the results are expressed as “not measured—n.m”. All analyzes were performed in triplicate.

### 2.5. Carbohydrate Profile (Mono-, Di- and Oligosaccharides)

The freeze-dried fractions were prepared, and the carbohydrate profile was determined according to [18]. An amount of 1 g of each fraction was diluted in 10 mL of ultra-pure water, homogenized in UltraTurrax (UltraTurrax IKA, T25, Werke, Germany) at 11,000 rpm 30 s^−1^ at room temperature and then centrifuged (4000× *g*, 5 min, 5 °C; Hettich Zentrifugen, model Rotanta 460R, Tuttlingen, Germany). The supernatant was filtered with a 0.22 µm regenerated cellulose membrane filter.

Carbohydrates were identified and quantified by high-performance anion-exchange chromatography coupled with a pulsed amperometric detection system (HPAEC-PAD) model DIONEX ICS-5000 (Thermo Fisher Scientific, Waltham, MA, USA). The flow rate was 1.0 mL·min^−1^, the column temperature was maintained at 30 °C, and the injection volume was 25 μL.

A chromatographic column (Carbopac PA1 250 × 4 mm, 10 μm particle size) was used to determine the monosaccharides, disaccharides and polyols (glucose, fructose, sucrose, maltose, cellobiose, raffinose, arabinose, verbascose, stachyose, xylitol and sorbitol). The isocratic mobile phase with 60% A (0.2 M NaOH) and 40% C (ultra-pure water) was used for 25 min. Afterward, 100% A was used for 5 min for column cleaning. Then, for a new run, column stabilization was performed using 60% A and 40% C.

A column (CarboPac PA100, 250 × 4 mm, 8.5 µm particle size) was used to analyze fructo- and malto-oligosaccharides (1-kestose (GF2), nystose (GF3) and 1-fructofuranosylnystose (GF4), maltotriose (G3), maltotetraose (G4), maltopentaose (G5), maltohexaose (G6) and maltoheptaose (G7). The gradient was performed as follows: 0–2 min, 48.5% A, 1.5% B (1 M sodium acetate containing 0.2 M NaOH); 2–18 min, 48.5–30% A and 1.5–20% B; 18–25 min, 100% B; and 25–30 min, 48.5% A and 1.5% B.

Carbohydrates were quantified using a linear calibration curve for carbohydrate standards (glucose, fructose, sucrose, G3, G4, G5, G6 and G7 from Sigma-Aldrich, St. Louis, MO, USA) and GF2, GF3 and GF4 (Wako Pure Chemical Industries, Osaka, Japan). All standards presented purity grade ≥98%. The carbohydrate contents of the samples were expressed in mg·g^−1^.

### 2.6. Minerals

The contents of iron (Fe), zinc (Zn), calcium (Ca), magnesium (Mg), potassium (K), manganese (Mn) and copper (Cu) were evaluated according to [19]. After mineralization with nitric acid (7%, analytical grade; Sigma-Aldrich, St. Loius, MO, USA) and hydrogen peroxide (2 mL, 30%; Synth, Diadema, Brazil), the samples were digested, and flame atomic absorption spectrophotometry (FAAS, Analyst 200, PerkinElmer, Waltham, MA, USA) was used to determine the mineral content. FAAS was used in absorption mode with a deuterium lamp to correct background radiation and with hollow cathode lamps to determine Fe (248.3 nm), Ca (422.67 nm), Mg (285.21 nm), Mn (279.48 nm), Cu (324.75 nm) and Zn (213.86 nm) contents. The K content was determined with FAAS in emission mode at 766.5 nm. Standard solutions of Fe, Ca, Mg, K, Mn, Cu and Zn (Sigma-Aldrich) at a concentration of 1000 mg·g^−1^ were used to construct the analytical curves.

### 2.7. Bioactive Compounds and Antioxidant Capacity

#### 2.7.1. Extract Preparation

The extract of all freeze-dried and ground fractions was obtained according to [20], with some modifications. An amount of 1.0 g of the samples was homogenized in UltraTurrax (UltraTurrax IKA, T25, Werke, Germany) with 15 mL of ethanol–acetone–water solution (7:7:6, *v*/*v*/*v*). This solution was mixed in an ultrasonic bath (UNIQUE, model UCS-2850, 25 kHz, 120 W, Brazil) for 30 min at room temperature and then centrifuged at 4000× *g* for 5 min at 5 °C (Hettich Zentrifugen, model Rotanta 460R, Tuttlingen, Germany). The supernatants were collected and reserved. This procedure was repeated twice more. Subsequently, the supernatants were collected and concentrated under vacuum at 35 °C (Rotavapor model RII, Büchi Labortechnik, Flawil, Switzerland) to remove the organic solvents and suspended in 10 mL of water.

#### 2.7.2. Phenolic Compounds Profile

The analysis of phenolic compounds profile was performed by ultra-high performance liquid chromatography (UHPLC) using an Acquity chromatograph coupled with a TQD Acquity mass spectrometer (Micromass-Waters, Manchester, UK) with electrospray ionization (ESI) and using a C18 BEH Waters Acquity column (2.1 mm × 50 mm × 1.7 µm particle size). A specific chromatographic method for the samples was developed according to [21], with modifications. Phase A was composed of acidic water (0.1% formic acid) and phase B of acetonitrile (HPLC grade). The gradient started at 5% A and 90% B; it increased to 50% B in 7.5 min, then to 100% B in 8.0 min, keeping these concentrations for 1.0 min and returning to the initial conditions in 9.1 min until 10 min. The conditions of the ESI analysis in negative mode were: capillary voltage of −3.00 kV, cone of −30.00 V, source temperature of 150 °C and desolvation temperature of 350 °C. In the tandem mass spectrometry analysis (MS-MS), used to examine the fragmentation of ions, a Collision-Induced Dissociation (CID) was performed at 20 V of collision energy. Initially, a full scan (*m*/*z* 100 to 1000) was carried out to check whether phenolics were present compared to a panel of standards. Finally, the analysis of the samples and the calibration curve of the standards in selected ion mode (SIM) were performed at *m*/*z* 191 (quinic acid), *m*/*z* 153 (protocatechuic acid), *m*/*z* 353 (chlorogenic acid), *m*/*z* 289 (catechin/epicatechin), *m*/*z* 163 (ρ-coumaric acid), *m*/*z* 609 (rutin), *m*/*z* 223 (synaptic acid), *m*/*z* 193 (ferulic acid) and *m*/*z* 447 (quercetrin).

#### 2.7.3. Total Phenolics Compounds (TPC)

This assay was performed according to [22], with some modifications. The sample solubilized in ethanol was added to 25 mL of the aqueous solution of the reagent Folin–Ciocauteal at 10% and to 2.0 mL of sodium carbonate at 7.5% and then incubated in water-bath for 6.0 min at 45 °C for development of the color. The absorbance was measured in a spectrophotometer (Beckman, model DU600, Fullerton, CA, USA) at 760 nm compared to a blank. To quantify the total phenolic compounds, a curve of gallic acid standard was built, and the results are expressed in mg of gallic acid equivalent·g^−1^ of freeze-dried sample (mg GAE·g^−1^ fdw).

#### 2.7.4. Total Flavonoid Content (TFC)

The total flavonoid content was determined using the method proposed by [23], through sample reactions with NaNO_2_, AlCl_3_ and NaOH, followed by absorbance reading on a spectrophotometer (Beckman, model DU600, Fullerton, CA, USA) at 510 nm. The quantification of total flavonoids of the samples was performed using a standard curve prepared with catechin and expressed as catechin equivalents. The final result is expressed in mg of catechin equivalents·g^−1^ of freeze-dried sample (mg CE·g^−1^ fdw).

#### 2.7.5. DPPH Scavenging Assay

The assay was performed using 200 µL of the sample with 1000 µL of the DPPH^•^ solution (0.004% *w*/*v*) according to [24], with some modifications. Both the sample and the standard curve were incubated for 30 min and protected from light at room temperature. The absorbance of the remaining DPPH was measured at 517 nm against a blank on a spectrophotometer (Beckman, model DU600, Fullerton, CA, USA). The results are expressed as micromoles of Trolox equivalents·g^−1^ of freeze-dried sample (μmol TE·g^−1^ fdw).

#### 2.7.6. Trolox Equivalent Antioxidant Capacity (TEAC) Assay

The total antioxidant capacity was determined through the test with ABTS^•+^, obtained by reacting 5.0 mL of ABTS 7 mM with 88 μL of 140 mM potassium persulfate (final concentration of 2.45 mM), according to the method described by [25]. The system allowed resting at room temperature for 12 to 16 h in the absence of light. Once ABTS^•+^ was formed, it was diluted with distilled water until an absorbance value of 0.700 ± 0.02 at 734 nm was obtained. The absorbance reading was obtained from the reaction of 50 μL of sample and 250 μL of ABTS^•+^ solution against the 734 nm blank in a microplate reader. The results are expressed as micromoles of Trolox equivalents·g^−1^ of freeze-dried sample (μmol TE·g^−1^ fdw).

#### 2.7.7. Oxygen Radical Absorbance Capacity—Hydrophilic Fraction (ORAC_HF_) Assay

The assays were performed according to the method described by Prior et al. [26]. A dark microplate of polystyrene with 96 wells was used. The volume of 20 µL of sample blank or Trolox standard plus 120 µL of fluorescein and 60 µL of AAPH [2,2′-azobis (2′-metilpropionamidine) dihydrochloride] was added to each well. The temperature was maintained at 37 °C for 80 min. Fluorescence was determined and recorded every minute on a NovoStar Microplate reader (New Brunswick Scientific Classic Series, model C76, Offerburg, Germany) with fluorescence filters (excitation and emission wavelengths of 485 and 520 nm, respectively). Trolox was used to prepare the standard curve, and the results are expressed as micromoles of Trolox equivalents·g^−1^ of freeze-dried sample (μmol TE·g^−1^ fdw).

### 2.8. Analysis of Volatile Organic Compounds (VOCs) in GC-MS

#### 2.8.1. Sample Conditions and SPME Extraction

Buritirana samples remained frozen at −40 °C for 36 months. After this period, they were unfrozen and pulped manually. The mass of 500 mg of the edible fraction (PU) was ground and homogenized with ultra-pure water (1:4, *w*:*v*) in 20 mL flasks with a screw cap containing a Teflon-coated septum for VOCs headspace microextraction [27]. SPME extractions were performed from the headspace of the samples according to the following conditions: DVB/CAR/PDMS fiber; equilibrium time of 10 min; extraction time of 15 min; and extraction temperature of 50 °C.

#### 2.8.2. GC-MS Analysis Procedure

The compounds were separated using an Agilent 7890A gas chromatography system (Agilent Technologies, Santa Clara, CA, USA) equipped with a GC DB-WAX column (30 m × 0.25 mm × 0.15 μm) and Agilent 5975C inert MSD with Triple-Axis Detector, using He as a carrier gas. The VOCs were desorbed for 5 min by inserting the SPME fiber into a GC injector (270 °C). The GC oven was programmed to maintain a temperature of 70 °C for 1 min, then to increase to 140 °C at a rate of 3 °C·min^−1^, and finally to 210 °C at a rate of 5 °C·min^−1^. The column flow rate was 1.0 mL·min^−1^. The MS was scanned in the range of 45–650 amu at 70 eV. The total analysis time was 39.33 min. Compounds were identified using NIST 14.0 database, and Linear Retention Index (LRI) was calculated with a series of n-alkanes (C7–C40).

### 2.9. Antibacterial Activity

#### 2.9.1. Micro-Organism and Culture Condition

The bacterial strains used in this study were *Escherichia coli* ATCC 10231, *Staphylococcus aureus* ATCC 6538, *Bacillus subtilis* ATCC 5061, *Bacillus cereus* ATCC 10876, *Salmonella choleraesuis* ATCC 10708 and *Peseudomonas aeruginosa* ATCC 13388. All strains were periodically harvested in Mueller–Hinton medium (Kasvi) (bacteria, 37 °C for 1 day) and stored under refrigeration conditions.

#### 2.9.2. Determination of Minimum Inhibitory Concentration (MIC)

Extracts of PU, PE, WS and SE fractions were used to assess antimicrobial susceptibility using the broth microdilution method according to CLSI [28]. These methods assessed the ability to inhibit bacterial growth by evaluating different known concentrations in a 96-well microplate. Initially, the compounds were transferred into the first well, and serial dilutions were made in the range of 4.0–0.001 mg·mL^−1^. Streptomycin sulfate (Sigma-Aldrich^®^) was used in the range of 0.5–0.0039 mg·mL^−1^ as the reference antibiotic control.

The antibacterial activity was carried out using Mueller–Hinton broth (Kasvi). The bacteria strains were standardized in Mueller–Hinton broth to 10^6^ CFU·mL^−1^. The inoculum was added to all wells, and the plates were incubated at 37 °C for 24 h. The MIC was defined as the lowest concentration of the sample that inhibited visible growth. As indicated by 2,3,5-triphenyltetrazolium chloride staining, dead cells did not stain.

### 2.10. Experimental Design and Statistical Analysis

A completely randomized design (DIC) was used for the experiment, the data were analyzed by ANOVA and, when differences were detected, the Tukey test was used for multiple means comparison using a significance level of 5%. The Principal Component Analysis (PCA) was used to understand possible variable–variable and variable–samples relations. The PCA was performed on standardized data to avoid the effect of different magnitude levels of response variables. Statistical analyses were carried out using the R 4.0.2 (2020) and the package FactorMineR 1.32 for exploratory multivariate data analysis. All analyses were performed in triplicate, and the results are expressed as mean ± standard deviation.

## 3. Results and Discussion

### 3.1. Biometric Characteristics

The buritirana fruits evaluated were oval drupes with not very hard peels similar to small overlapping fish scales light orange in color. The seed is covered by a fibrous layer and a lemon-yellow pulp with a characteristic odor.

The whole fruit had a mass of 10.07 g (Table 1). The buritirana fruit showed a lower mass in comparison with buriti (*Mauritia vinífera*) [29], which presented a WS fraction of 60.43 g, PU of 13.55 g, PE of 15.68 g and SE of 31.19 g. Moreover, the buritirana seed (Table 1) presented the highest mass, followed by the peel and pulp (6.46 g > 1.94 g > 1.67 g), representing 64.15%, 19.27% and 16.58% of the fruit, respectively. These values were higher than those of buriti pulp (*Mauritia flexuosa*) grown in Goiás and Pará (16.43% and 8.53%, respectively) [30].

The length (29.20 mm) and the apical (19.30 mm), central (24.75 mm) and basal (21.25 mm) widths indicate that the buritirana fruit had an oblong shape. Ruiz and Villena [11] evaluated the buritirana fruit grown in Iquito, a region of Loreto, Peru, and found similar results for length (25–35 mm) and width (20–30 mm).

### 3.2. Proximate Composition, Physicochemical Characteristics and Vitamin C of Buritirana Fractions

The seed presented the highest moisture (Table 2) in comparison with the other fractions (*p* < 0.05). The amount of total carbohydrates was the highest (*p* > 0.05) in SE and PE fractions (23.24% for both), followed by the WS (17%) and PU (8.06%) fractions. The PU and WS fractions differ statistically and showed the highest amounts of lipids (20.20% and 16.57%, respectively), followed by the PE (13.43%) and SE (0.27%) fractions. These results are similar to those found by Souza et al. [12]

For proteins, the PU and SE fractions showed similar values (5.96% for both, *p* < 0.05), followed by WS and PE fractions (5.53% and 5.34%, respectively). The ash content varied from 1.58 g ± 0.03 (PU) to 3.77 g ± 0.02 (SE) (*p* < 0.05). The WS, PE and PU fractions showed the highest total energy, (381.74 Kcal·100 g^−1^, 368.78 Kcal·100 g^−1^, 381.88 Kcal·100 g^−1^, respectively; *p* < 0.05) followed by the SE fraction (280.72 Kcal·100 g^−1^).

There was no difference between PU and PE fractions (*p* > 0.05). The highest energy value of the WS and PE fractions can be explained by the fact they also have the highest lipid amount, since lipids have 9 Kcal·g^−1^ and proteins and carbohydrates only have 4 Kcal·g^−1^. The total energy of the PU fraction corresponds to ~7 portions (50 g of dehydrated buritirana pulp), 18.43% of the recommended daily intake, 2000 Kcal for adults [31].

The PU fraction showed the highest soluble fiber content with (10.53 ± 1.97 g·100 g^−1^), which was similar (*p* > 0.05) to those of the WS (10.33 ± 0.68 g·100 g^−1^) and PE (8.04 ± 2.26 g·100 g^−1^) fractions. The lowest soluble fiber content was found in the SE fraction (5.56 ± 0.14 g·100 g^−1^, *p* < 0.05). Fiber consumption is related to fecal volume, viscosity and fermentation, which may contribute to the reduction in hyperglycemia, hypertension and oxidative stress in people with type 2 diabetes [32,33]. This indicates that the buritirana fruit is rich in dietary fiber and can be used to treat chronic diseases associated with the intestine. 

The concentrations of vitamin C, in decreasing order, were: PE (242.45 mg·100 g^−1^) > WS (223.53 mg·100 g^−1^) > PU (205.00 mg·100 g^−1^) > SE (106.33 mg·100 g^−1^). However, there were no statistical differences between the WS and PE fractions. Buritirana fractions were revealed to be a great source of vitamin C compared to several Brazilian tropical fruits [34]. Additionally, the vitamin C in the peel of buritirana represents about 12% of the vitamin C content of acerola (*Malpighia emarginata*), which after camu-camu (*Myrciaria dubia*), is considered one of the fruits with the highest concentration of vitamin C among Brazilian tropical fruits [35,36].

Regarding pH, the seed was the only one (*p* < 0.05) that presented low acidity (pH 5.88), while the other fractions can be considered very acidic (pH < 4.0). Acidic fruits are widely used for the preparation of sweets and jellies, as they do not require the addition of organic acids to reach the optimum gelation point. The TSS reflects the organic acids, vitamins, minerals and total soluble sugars in a fruit, and is usually represented by the total soluble carbohydrates. The TSS value in the WS fraction was 10.48 g and 8.18 g in the PU fraction. The TTA was 1.28 ± 0.00 and 1.71 ± 0.04 for WS and PU and resulted in a TSS/TTA ratio of 8.18 ± 0.08 and 4.83 ± 0.06, respectively, with an emphasis on the WS fraction. The ratio is related to the flavor attribute and indicates the balance between sugars and organic acids; it is considered an important parameter to determine the ripeness and palatability of the fruits [33].

### 3.3. Carbohydrate Profile: Mono-, Di- and Oligosaccharides

Total carbohydrates were investigated and classified and quantified as mono-, di- and oligosaccharides as shown in Table 1. Among monosaccharides, glucose was the main simple sugar found in all fractions, and the PU fraction presented the highest value at 1687.87 ± 13.50 mg·100 g^−1^ (*p* < 0.05). The SE fraction showed the highest fructose content (801.67 ± 4.04 mg·100 g^−1^), about 1.8, 1.5, 2.0 times greater than that of the WS, PU and PE fractions, respectively. The values of glucose and fructose were lower than those found in buriti (*Mauritia flexuosa*) (13,240 mg·100 g^−1^ for glucose and 2770 mg·100 g^−1^ for fructose) but higher than those found in açaí (*Euterpe oleraceae*) (250 mg·100 g^−1^ for glucose and 90 mg·100 g^−1^ for fructose) [4]. Regarding the disaccharides, sucrose was found only in the seed (3038.37 ± 28.76 mg·100 g^−1^). The sucrose concentration found in this study was higher than that found in the uvaia seed (1352 ± 0.10 mg·100 g^−1^) [37]. The maltose was found in all fractions and ranged from 466.80 ± 21.52 mg·100 g^−1^ in the PU to 523.32 ± 23.87 mg·100 g^−1^ in the SE; therefore, there was no statistical difference among the fractions (*p* > 0.05). The maltose content of the WS, PU, PE and SE fractions was higher than that of the edible fractions and seeds of uvaia (*Eugenia pyriformis*) and araçá-boi (*Eugenia stipitata*) [27,37]. The buritirana has a high concentration of maltose, which represents about 50% of the maltose content in adulterated apple juices [38]. Maltose is a very important fermentable sugar, with potential to stabilize an emulsion in cooling and freezing processes, and when hydrolyzed it turns into glucose, which is widely used to reduce sweetness and increase the brightness of base products of fruits, such as jellies, among others [39,40].

Malto-oligosaccharides were not found in any of the fractions evaluated. Fructo-oligosaccharides were found in the seed, generally GF2 and GF3, and was approximately 16.7 and 2.0 times higher than in the WS fraction, respectively; GF2 and GF3 concentrations in WS and SE fractions were 157.91 ± 10.21 mg·g^−1^ and 2646.49 ± 137.65 mg·g^−1^, respectively (Table 2). This is the first study that includes an analysis of the carbohydrate profile of the buritirana fruit. Other studies on native fruits from different regions of Brazil have shown the presence of the following compounds: GF2 (63.80 mg·100 g^−1^) and GF3 (374.91 mg·100 g^−1^) in the juá-açu pulp (*Solanum oocarpum* Sendtn.); GF2 (26.81 mg·100 g^−1^) and GF3 (66.69 mg·100 g^−1^) in the fruta-do-lobo pulp (*Solanum lycocarpum* St. Hill) [33]; GF2 (27 ± 0.03 mg·100 g^−1^) in the edible fraction of *Eugenia stipitata* [27]; and GF2 (21 ± 0.04 mg·100 g^−1^) in the uvaia seed (*Eugenia pyriformis*) [37]. Among the reported fruits, the juá-açu pulp showed the highest levels of GF2 and GF3. However, in comparison to the juá-açu seed, the value of GF2 (2646.49 mg·100 g^−1^) of the buritirana seed was 41.5 times higher, and the value of GF3 was only 1.8 times lower. Oligosaccharides are specific groups of carbohydrates that have prebiotic activity and are applied as food ingredients, additives for cosmetics, pharmaceutical products and preservatives for fruits and vegetables. They are associated with several functional properties, such as improvement of intestinal function, mineral absorption, regulation of lipid and glycemic metabolism and reduction in the risk of developing colon cancer [27,41]. This reinforces the importance of investigating these compounds and shows that buritirana has great potential for the production of food products with functional attributes.

### 3.4. Mineral Content

All fractions of buritirana have nutritional potential in micro- and macrominerals (Table 2), but there were differences among the fractions (*p* < 0.05). The highest mineral contents in mg·100 g^−1^ were: Fe (3.58 ± 0.33, PE), Zn (2.23 ± 0.07, WS), Ca and Mn (65.189 ± 0.71 and 3.54 ± 0.08, PU) and Mg, K and Cu (112.96 ± 0.87, 800.01 ± 8.91 and 0.79 ± 0.00, SE). On the other hand, the lowest contents were: Fe, Zn and Mn (2.70 ± 0.13, 1.94 ± 0.03 and 2.15 ± 0.07, SE), Ca, Mg and K (34.46 ± 1.20, 43.96 ± 1.03 and 528.12 ± 23.36, PE) and Cu (0.43 ± 0.04, PU).

Fruit from the same family, buriti (*Mauritiaflexuosa*), açaí (*Euterpeoleraceae*) and macaúba (*Acrocomia aculeata*), were also studied regarding mineral content [4] by convention; the highest mineral content (mg·100 g^−1^) found here will be compared with those of these fruits. The content of Fe in buritirana was higher than that of buriti, but lower than that of macaúba and açaí; the Zn content was lower than that of macaúba and higher than that of buriti and açaí; the Mg content was lower than that of the three other fruits; the K content was lower than that of açaí but higher than that of buriti and macaúba; the Cu content was higher than that of buriti and macaúba (açaí, not reported); the Ca content was lower than that of the three fruits; and the Mn content was lower than that of açaí but higher than that of buriti and macaúba. Minerals are essential elements for good maintenance of the body, and according to [42], the consumption of 100 g of edible buritirana fractions (WS and PU) provides the recommended daily intake (RDA) of Ca for all lifestyle groups; Cu represents 50% of the RDA for infants, children, males and females >70 years); Fe provides about 26.4% of the RDA for children 0–8 years; Mg provides more than 50% of the recommendation for infants and children aged 7 months–3 years); Mn also supplies the RDA of all groups; and finally, Zn provides about 74% of the RDA for children aged 1–3 years) [42]. All fractions of the buritirana studied are sources rich of minerals for people of different lifestyles, sex and age, as they present more than the minimum of 15% of the recommended dietary intake per 100 g of sample [43]. PE and SE fractions, which are not edible, can be used as sources of minerals to enrich food.

**Figure 2:**
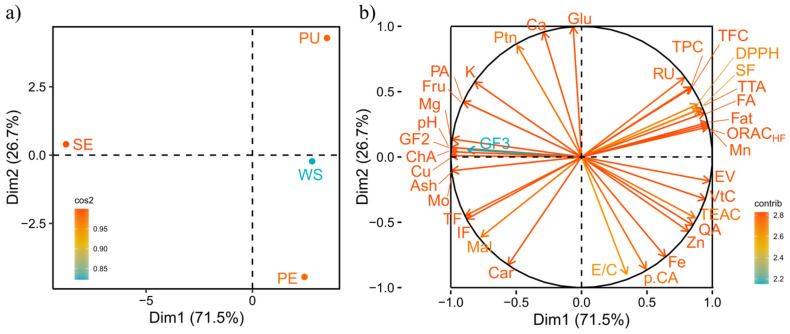
Graphical representation of the Principal Component Analysis (PCA) of the buritirana fruit fractions: (**a**) Fractions: WS: whole without seed; PU: pulp; PE: peel; and SE: seed. (**b**) Responses of proximate composition, carbohydrate profile, physicochemical characteristics, vitamin C content, bioactive compounds and antioxidant capacity; Mo: moisture; Ptn: protein; Car: total carbohydrates; Fat: total fat; VtC: vitamin C; TF: total fiber; IF: insoluble fiber; SF: soluble fiber; EV: Energy value; TTA: total titratable acidity; Glu: glucose; Fru: fructose; Suc: sucrose; Mal: maltose; GF2: 1-kestose; GF3: nystose; Ca: calcium; Mg: magnesium; Fe: iron; Zn: zinc; K: potassium; Cu: copper; Mn: manganese; pH: hidrogenionic potential.; TPC: total phenolic compounds; TFC: total flavonoids; TEAC: Trolox equivalent absorbances capacity; DPPH: DPPH scavenging; ORAC_HF_: oxygen radical absorbance capacity; QA: quinic acid; FA: ferulic acid; PA: protocatechuic acid; ChA: chlorogenic acid; E/C: epicatechin/catechin; p.CA: ρ-Coumaric acid; RU: rutin; QE: quercetrin.

Figure 2 shows a clear and concise interpretation of the possible relationships among the responses of proximate composition, carbohydrate profile, physicochemical characteristics, bioactive compounds, including antioxidant properties and vitamin C content, and the fractions. The first two main components explain 71.5% and 26.7% of the data variability, totaling 98.2%. This value was higher than that recommended by Abdi and Williams [44], who reported that the first selected main components must correspond to a minimum of 75% of the variability among the samples.

The SE fraction (Figure 2a) had greater values for Mo, Ash, TF, IF, Fru, Suc, Mal, K, Mg, Cu, GF2, GF3, pH, ChA and PA (Figure 2b) as they are in the same geometric space as the SE. Among the nine phenolic compounds investigated, six were present in the seed, and among these, two were below the LOQ (Table 3). Thus, since the phenolic compounds are directly related to the antioxidant capacity, it can be observed (Table 4) that in all the assays, SE presented lower antioxidant potential compared to the other fractions. On the other hand, oligosaccharides such as GF2 and GF3 were identified in a greater proportion in the buritirana seed and can contribute to the formulation of products with functional claim. Since these compounds have prebiotic action, they can regulate the gut microbiota and help to prevent and fight against diseases [45,46]. Therefore, the characteristics of this fraction can add value to a part of the buritirana fruit that is usually discarded, as occurs with most fruits that are consumed fresh or utilized in processing units to obtain their derivatives.

Similarly, the PU fraction (Figure 2a) was more associated with the highest values of the variables Ptn, Fat, Ca, Mn, SF, Glu, TTA, ORAC_HF_, TPC, TFC, FA, and RU (Figure 2b and Table 2). Thus, the pulp presented a high energy value and great antioxidant potential. On the other hand, the PE fraction (Figure 2a) was better represented by Car, Fe, Zn, VtC, EV, QA, TEAC, p.CA and E/C (Figure 2b and Table 2). The buritirana peel has potential to be used as a source of dietary fiber and natural antioxidants in food. Finally, the WS fraction, which is in light blue in Figure 2a, showed intermediate values when compared to the PU and PE fractions.

### 3.5. Bioactive Compounds and Antioxidant Potential of the Buritirana Fruit Fractions

#### 3.5.1. Phenolic Compounds Profile

The phenolic compound profile of the buritirana fruit fractions was determined using UHPLC-ESI-MS. This was the first time that the profile of phenolic compounds of the buritirana fruit fractions was evaluated, and they are shown in Table 3. Investigating phenolic compounds is paramount since they are non-nutritive ingredients synthesized by the secondary metabolism of plants and play an important role in human health, in addition to having several bioactive properties [4,47,48]. Nine compounds were identified and quantified: quercetrin, rutin, epicatechin/catechin, and ferulic, sinapic, p-coumaric, chlorogenic, protecatechuic and quinic acids. However, quercetrin and sinapic acids were below the limit of quantification (<LOQ). The quinic and protocatechuic acids and epicatechin/catechin were quantified in all fractions. They showed the highest contents among the acids; their content ranged from 0.03 ± 0.07 μg·mL^−1^ for SE (*p* < 0.05) to 6.77 ± 1.43 μg·mL^−1^ in the WS fraction. The PU fraction showed the highest content of rutin (1.14 ± 0.01 μg·mL^−1^) and ferrulic acid (0.05 ± 0.00 μg·mL^−1^) (*p* < 0.05).

*p*-Coumaric acid content was similar (*p* > 0.05) between fractions WS and PE (0.01 μg·mL^−1^) and was not determined in the PU and SE fractions. Phenolic compounds were also investigated in the oil of the buritirana fruit fractions (*Mauritiella armata*), and the authors reported that the fractions WS, PU and PE also presented phenolic compounds similar to those found in this study [12].

#### 3.5.2. Total Phenolic Compounds, Total Flavonoids and Antioxidant Capacity

The contents of total flavonoids and total phenolic compounds, as well as the antioxidant capacity of the buritirana fruit fractions, were evaluated by the DPPH, TEAC and ORAC_HF_ assays. The results are shown in Table 4.

The PU fraction showed the highest content of TPC and TFC and antioxidant capacity according to DPPH (10.60 ± 0.08 mg GAE·g^−1^, 0.74 ± 0.38 mg CE·g^−1^ and 234.25 ± 4.42 μmol TE·g^−1^, respectively), and the values differed statistically from those of WS, PE and SE fractions (*p* < 0.05). An antioxidant capacity of 140.75 ± 0.30 and 136.95 ± 0.26 μmol TE·g^−1^ according to DPPH was similar (*p* > 0.05) between the WS and PE fractions, respectively. For the TEAC assay, the highest (*p* < 0.05) antioxidant capacity was observed in the PE fraction (781.09 ± 4.32 μmol TE·g^−1^). On the other hand, the PU fraction showed the highest ORAC_HF_ values (2.12 ± 0.13 μmol TE·g^−1^), while the SE showed the lowest ORAC_HF_ values (0.50 ± 0.03 μmol TE·g^−1^). The values for TPC and TFC of the PU fraction were above those found by Araújo et al. [27], who reported values of 9.06 ± 0.42 mg GAE·g^−1^ and 1.25 ± 0.12 mg CE·g^−1^, respectively, for the edible fraction of *Eugenia stipitata*, a Brazilian fruit native to the Northeast region. According to the authors, this may be due to environmental variables, since the buritirana comes from the North region of Brazil. In another study on the *Eugenia pyriformis* (another Brazilian native fruit), the authors found a TEAC value (83.39 ± 0.79 μmol TE·g^−1^) about 5.4 times lower and 11.1 times higher than those found here for the PU and SE fractions, respectively [37]. Finally, the results found for the antioxidant capacity showed that the ORAC_HF_ values for the peel (2.66 ± 0.34 μmol TE·g^−1^), pulp (4.14 ± 0.18 μmol TE·g^−1^) and seed (3.16 ± 0.03 μmol TE·g^−1^) of the fruta-do-lobo (*Solanum lycocarpum* St. Hill) were approximately 1.7, 1.9 and 6.3 times higher, respectively, than those we found for the same fractions evaluated [33].

### 3.6. Volatile Organic Compounds and Antibacterial Activity

According to the conditions previously described, no VOCs were identified in the buritirana pulp, and the fractions tested did not show activity against the micro-organisms at any concentration evaluated. There are no reports in the literature that define the aroma and flavor of buritirana, not even among fruits of the same family, to compare their sensory characteristics. The buritirana in nature is not a fruit that has a pronounced aroma like other Brazilian fruits, but when prepared for different products, these sensory characteristics are remarkable. Therefore, according to [49], it can be said that buritirana has its own aroma and flavor. The storage time and temperature most likely have negatively influenced the chemical composition of the fractions, affecting the properties of bioactive compounds that are related to antioxidant and antibacterial activity, among others. Although freezing is widely used to prolong shelf life, this conservation technique can affect the chemical composition and quality of the fractions [9,50,51].

## 4. Conclusions

The WS and PU fractions showed physicochemical characteristics that indicate that the buritirana has great potential in fruit technology for new products with high added value.

The fractions of the buritirana fruit showed nutritional value since they contained proteins, carbohydrates, vitamin C, fibers (soluble and insoluble) and minerals (Fe, Zn, Ca, Mg, K, Mn and Cu). Therefore, buritirana could be an important source of nutrients, which could contribute to the economy of the local population, as well as to the sustainability of Brazilian fruits since they are widely utilized in the agroindustry. Although the buritirana seed is not an edible fraction, it contains interesting oligosaccharides (1-kestose and nystose) and a high sucrose content, which could be used as valuable ingredients in the food, cosmetic and pharmaceutical industries.

Higher levels of epicatechin/catechin and quinic and protocatechuic acids were found in all fractions of the ten identified phenolic compounds. The results of antioxidant assays associated with the highest concentrations of quinic acid and rutin showed that the WS and PU fractions had the best antioxidant potential, respectively. Thus, these results suggest that these fractions can be further studied in biological assays to demonstrate their functional potential.

Under the conditions described above, no VOCs were identified in the buritirana pulp, and none of the fractions showed inhibition potential against the investigated bacteria. Our study demonstrates the nutritional and functional potential of buritirana and can contribute to future investigations, exploration, propagation and commercialization of this species.

## Figures and Tables

**Table 1:** Biometric characteristics of the buritirana fruit and its fractions.

Biometric Characteristics (*n* = 20)		
Mass of whole fruit	10.07 g ± 1.02	
Mass of peel	1.94 g ± 0.13	* 19.27%
Mass of pulp	1.67 g ± 0.60	* 16.58%
Mass of seed	6.46 g ± 0.58	* 64.15%
Longitudinal length	29.20 mm ± 0.89	
Apical diameter	19.30 mm ± 1.13	
Central diameter	24.75 mm ± 1.52	
Basal diameter	21.25 mm ± 1.33	

* Percentage (*w*/*w*) of each fraction related the whole fruit.

**Table 2:** Proximate composition, saccharides, minerals and vitamin C contents, and physicochemical characteristics of the buritirana fruit fractions.

Characteristics		Fractions			
		WS	PU	PE	SE
Proximate composition units per 100 g	Moisture g	1.73 ± 0.13 c	1.58 ± 0.03 c	2.02 ± 0.11 b	3.77 ± 0.02 a
	Total carbohydrates g	17.00 ± 0.75 b	8.06 ± 0.15 c	23.24 ± 0.90 a	23.24 ± 0.88 a
	Total fat g	16.57 ± 0.10 b	20.20 ± 1.98 a	13.43 ± 1.01 c	0.27 ± 0.03 d
	Protein g	5.53 ± 0.14 b	5.96 ± 0.09 a	5.34 ± 0.05 b	5.96 ± 0.27 a
	Ash g	1.73 ± 0.13 c	1.58 ± 0.03 c	2.02 ± 0.11 b	3.77 ± 0.02 a
	Total fiber g	71.22 ± 0.98 b	65.46 ± 2.41 c	73.35 ± 1.12 b	80.74 ± 0.14 a
	Soluble fiber ^1^ g	10.33 ± 0.68 a	10.53 ± 1.97 a	8.04 ± 2.26 ab	5.56 ± 0.14 b
	Insoluble fiber g	60.89 ± 0.30 c	53.32 ± 1.58 d	65.32 ± 1.15 b	75.18 ± 0.00 a
	Total energy value Kcal	381.74 ± 4.16 a	368.78 ± 19.20 a	381.88 ± 3.70 a	280.72 ± 3.93 b
Monosaccharides units per 100 g	Glucose mg	1228.00 ± 34.00 b	1387.87 ± 13.50 a	1034.23 ± 23.60 c	1256.00 ± 4.20 b
	Fructose mg	453.00 ± 46.00 c	547.50 ± 5.30 b	390.63 ± 10.28 c	801.67 ± 4.04 a
Disaccharides units per 100 g	Sucrose mg	n.d	n.d	n.d	3038.37 ± 28.76 a
	Maltose mg	479.00 ± 11.00 a	466.80 ± 21.52 a	509.97 ± 27.82 a	523.32 ± 23.87 a
Oligosaccharides units per 100 g	1-Kestose (GF2) mg	157.91 ±10.21 b	n.d	n.d	2646.49 ±137.65 a
	Nystose (GF3) mg	106.45 ± 4.57 b	n.d	n.d	209.15 ± 12.08 a
Minerals units per 100 g	Fe mg	3.29 ± 0.26 a,b	2.88 ± 0.15 b	3.58 ± 0.33 a	2.70 ± 0.13 b
	Zn mg	2.23 ± 0.07 b	2.15 ± 0.02 b	2.38 ± 0.04 a	1.94 ± 0.03 c
	Ca mg	51.46 ± 2.82 c	65.189 ± 0.71 a	34.46 ± 1.20 d	60.05 ± 0.60 b
	Mg mg	46.17 ± 0.66 c	49.12 ± 1.47 b	43.96 ± 1.03 c	112.96 ± 0.87 a
	K mg	608.67 ± 9.04 c	672.64 ± 39.80 b	528.12 ± 23.36 d	800.01 ± 8.91 a
	Mn mg	3.37 ± 0.10 a	3.54 ± 0.08 a	3.08 ± 0.11 b	2.15 ± 0.07 c
	Cu mg	0.44 ± 0.02 b	0.43 ± 0.04 b	0.45 ± 0.03 b	0.79 ± 0.00 a
Vitamin units per 100 g	Vitamin C mg	223.53 ± 11.164 a,b	205.00 ± 11.16 b	242.45 ± 10.08 a	106.33 ± 10.71 c
Physicochemical characteristics units per 100 g	Total titratable acidity (g citric acid)	1.28 ± 0.00 b	1.71 ± 0.04 a	1.05 ± 0.07 c	0.25 ± 0.02 d
	pH	3.36 ± 0.00 b	3.37 ± 0.00 b	3.37 ± 0.00 b	5.88 ± 0.02 a
	Total soluble solids g	10.48 ± 0.08 a	8.18 ± 0.08 b	n.m	n.m
	Ratio TSS/TTA	8.18 ± 0.08 a	4.83 ± 0.06 b	n.m	n.m

^1^: Soluble fibers were calculated as the difference between total fiber and insoluble fibers. TSS: total soluble solids; TTA: total titratable acidity; WS: whole without seed; PU: pulp; PE: peel; SE: seed. Averages with different letters in the line indicate statistical differences according to Turkey test (*p* < 0.05). n.d: not detected; n.m: not measured. *n* = 3. Season: November.

**Table 3:** Phenolic compounds found by UHPLC-ESI-MS in the buritirana fruit fractions.

			Fractions			
Phenolic Compounds	Mass [M − H]	RT (Min.)	WS	PU	PE	SE
			Concentration (μg·mL^−1^)			
Quercetrin	447	3.90	n.m	<LOQ	<LOQ	<LOQ
Ferulic acid	193	3.60	0.04 ± 0 b	0.05 ± 0 a	0.03 ± 0.01 c	n.m
Sinapic acid	223	3.50	<LOQ	n.m	<LOQ	<LOQ
Rutin	609	3.40	0.61 ± 0 b	1.14 ± 0.01 a	0.37 ± 0.01 c	n.m
*p*-Coumaric acid	163	3.25	0.01 ± 0 a	n.m	0.01 ± 0 a	n.m
Epicathecin/Cathecin	289	2.70	0.03 ± 0 b	0.01 ± 0 c	0.10 ± 0 a	0.01 ± 0 c
Chlorogenic acid	353	2.25	0.06 ± 0 b	n.m	0.10 ± 0 b	2.51 ± 0.1 a
Protecatechuic acid	153	1.75	0.06 ± 0 c	0.08 ± 0 b	0.04 ± 0 d	0.14 ± 0.01 a
Quinic acid	191	0.70	6.77 ± 1.43 b	5.20 ± 0.11 c	8.25 ± 0.24 a	1.91 ± 0.07 d

RT: retention time; LOQ: limits of quantification; WS: whole without seed; PU: pulp; PE: peel; SE: seed; Average with different letters in the line indicate statistical differences according to Turkey (*p* < 0.05). n.m: not measured; quercetrin LOQ: 0.88 μg·mL^−1^; sinapic acid LOQ: 0.43 μg·mL^−1^.

**Table 4:** Content of total flavonoids, content of total phenolic compounds and antioxidant capacity of the buritirana fruit fractions.

Parameters	Fractions			
	WS	PU	PE	SE
TPC ^1^	8.51 ± 0.14 b	10.60 ± 0.08 a	4.70 ± 0.10 c	1.54 ± 0.03 d
TFC ^2^	0.53 ± 5.97 b	0.75 ± 0.38 a	0.29 ± 0.43 c	0.02 ± 0.07 d
DPPH ^3^	140.75 ± 0.30 b	234.25 ± 4.42 a	136.95 ± 0.26 b	24.35 ± 0.50 c
TEAC ^3^	743.02 ± 9.94 b	448.40 ± 9.83 c	781.09 ± 4.32 a	38.44 ± 1.70 d
ORAC_HF_^3^	1.82 ± 0.01 b	2.12 ± 0.13 a	1.55 ± 0.17 b	0.50 ± 0.03 c

WS: whole without seed; PU: pulp; PE: peel; SE: seed; ^1^: result expressed as mg GAE·g^−1^ fdw (freeze-dried sample); ^2^: result expressed as mg CE·g^−1^ fdw; ^3^: result expressed as μmol TE·g^−1^ fdw; Averages with different letters in the line indicate statistical differences according to Turkey (*p* < 0.05).

## Data Availability

The data presented in this study are available on request from the corresponding author.
